# Effect of topical ozonetherapy on gingival wound healing in pigs: histological and immuno-histochemical analysis

**DOI:** 10.1590/1678-7757-2018-0015

**Published:** 2018-12-10

**Authors:** Zeynep Tastan Eroglu, Bulent Kurtis, Hasan Ayberk Altug, Sermet Sahin, Gulay Tuter, Emre Baris

**Affiliations:** 1 Hospital of Oral and Dental Health, Konya, Turkey.; 2 Gazi University, Faculty of Dentistry, Department of Periodontology, Ankara, Turkey.; 3 University of Health Sciences, Gulhane Faculty of Dentistry, Department of Oral and Maxillofacial Surgery, Ankara, Turkey.; 4 Biruni University, Faculty of Dentistry, Department of Periodontology, İstanbul, Turkey.; 5 Gazi University, Faculty of Dentistry, Department of Oral Pathology, Ankara, Turkey.

**Keywords:** Ozone, Transforming growth factor beta (TNF-β), Vascular endothelial growth factor (VEGF)

## Abstract

In this study, the effects of ozonetherapy on secondary wound healing were evaluated histologically and immuno-histochemically. Material and Methods: 8 healthy pigs were used in this study. Six wounds with 10 mm in diameter were created through the punch technique on the palatinal gingiva of each pig. Ozone gas was applied on only 3 wounds (test group) and the remaining 3 were left to natural healing (control group). Biopsy samples were taken from one of the wounds in each group on the third day, from another wound of each group on the seventh day, and from another one on the tenth day. Routine histological analysis and immuno-histochemical staining were performed to investigate transforming growth factor-beta (TGF-β) and (VEGF) expressions. Results: No statistical difference was found between the test and control groups in terms of collagen fibers, epithelial formation and inflammation scores. A VEGF expression found in the test group was statistically higher than control group samples taken on the 3^rd^ and 7^th^ day. There was no statistical difference between the test and control groups in terms of TGF-β expression on any of the sampling days. Conclusion: The topical application of ozone gas could be effective in the early stages of wound healing by increasing the amount of VEGF expression. Clinical Relevance: Topical application of ozone gas may be effective in the early stages of oral wound healing.

## Introduction

Oral wound healing is a dynamic and complex process of restoring cellular structures and tissue layers ^1^ . During this process, coordination occurs among epithelial cells, platelets, endothelial cells, fibroblasts and macrophages ^2^ .

Wound healing occurs in overlapping phases, which are inflammation, re-epithelialization, granulation tissue formation, matrix formation, and tissue remodeling ^2^ .

Re-epithelialization or epithelial healing is the most important and most complex of these processes. It includes cellular movement, proliferation and differentiation that provide functional and structural tissue repair ^3^ .

Secondary healing following surgical procedures, such as free gingival autograft, laterally sliding flap, gingivectomy and gingivoplasty, is always slower than primary healing.

The oral cavity provides a unique environmental challenge for healing wounds produced during various periodontal surgical procedures. Trauma from mastication, relatively large commensal oral flora and elevated levels of dental plaque can impair the normal sequence of the healing process ^4^ ^,^ ^5^ . Therefore, there is concern regarding the delayed healing of oral cavity wounds ^2^ .

In previous studies, it was observed that antimicrobials such as hydrogen peroxide, chlorhexidine, sodium hypochlorite, and povidone iodine cause cytotoxic effects on epithelial cells and gingival fibroblast cells ^6^ ^,^ ^7^ .

Another alternative agent that has recently been used in dentistry because of its effect on wound healing is ozone.

Ozone (also known as triatomic oxygen and trioxygen) is an allotropic form of oxygen occurring naturally in the Earth's atmosphere. It is created in nature when ultraviolet rays cause oxygen atoms to temporarily recombine in groups of three ^8^ .

It is an unstable gas and it quickly gives up nascent oxygen molecules to form oxygen gas. Due to its property of releasing nascent oxygen, it has long been used in human medicine to kill bacteria and fungi, to inactivate viruses and to control hemorrhages ^8^ .

Moreover, under the influence of ozone, improved rheological properties, activated cellular metabolism, raised intracellular ATP concentrations and expressions of cytokines relevant to wound healing, especially transforming growth factor-β1 (TGF-β1), have been observed ^1^ ^,^ ^9^ .

Medical grade ozone is produced commercially in ozone generators and these machines provide the topical administration of the ozone gas ^8^ . Because ozone gas has increased growth factors, activated local antioxidant mechanisms, quenched oxidant activity and promoted tissue repair, the application of ozone gas on the wound may accelerate wound healing ^2^ ^,^ ^10^ . However, there is no consensus or direct evidence that ozone plays a central role in the healing process. In addition, according to our knowledge, there is no histological or immuno-histochemical study that evaluated the topical effects of the ozone gas on wounds in the gingival region.

This study was designed to evaluate the therapeutic effect of topical ozone gas on gingival wound healing in the pig model, using histological and immuno-histochemical analysis, and to elucidate its therapeutic mechanisms that are associated with transforming growth factor- β (TGF-β) and vascular endothelial growth factor (VEGF).

## Material and methods

This controlled, randomized animal trial was carried out at Gulhane Military Medical Academy (GMMA) (University of Health Sciences), Gulhane Experimental Animal Research Center and Gazi University Faculty of Dentistry Department of Oral Pathology Laboratory, and approved by the GMMA Animal Experiments Ethics Committee (Ethics Code: Etik-2012-64)

Eight adult, healthy pigs were used, weighing between 40-60 kg. The study was designed to create both experimental and control wounds on the same animal, and tissue samples were taken on days 3, 7 and 10 for histological and immuno-histochemical analysis.

### Surgical procedures

The pigs were anesthetized with an intramuscular injection of ketamine hydrochloride (6-10 mg/kg), xylasin hydrochloride (1-3 mg/kg) and inhalation of isoflurane (1.5-3 mg/kg) before surgical procedures.

Impressions were taken from the palatal region of each pig with silicone impression material (Zeta Plus^®^, Zhermack, Italy) before creating the wound in the palatal areas. Six perforations were created on the silicone impression by using a punch biopsy method (10 mm in diameter) to facilitate the glass probe of ozone device localization. Individual silicone impressions were again placed on the palatal region, and the palatal mucosa was marked with a sterile punch biopsy knife (Paramount Sterile Dermal Biopsy Punch, Germany) through the silicone perforations according to projections.

**Figure 1:**
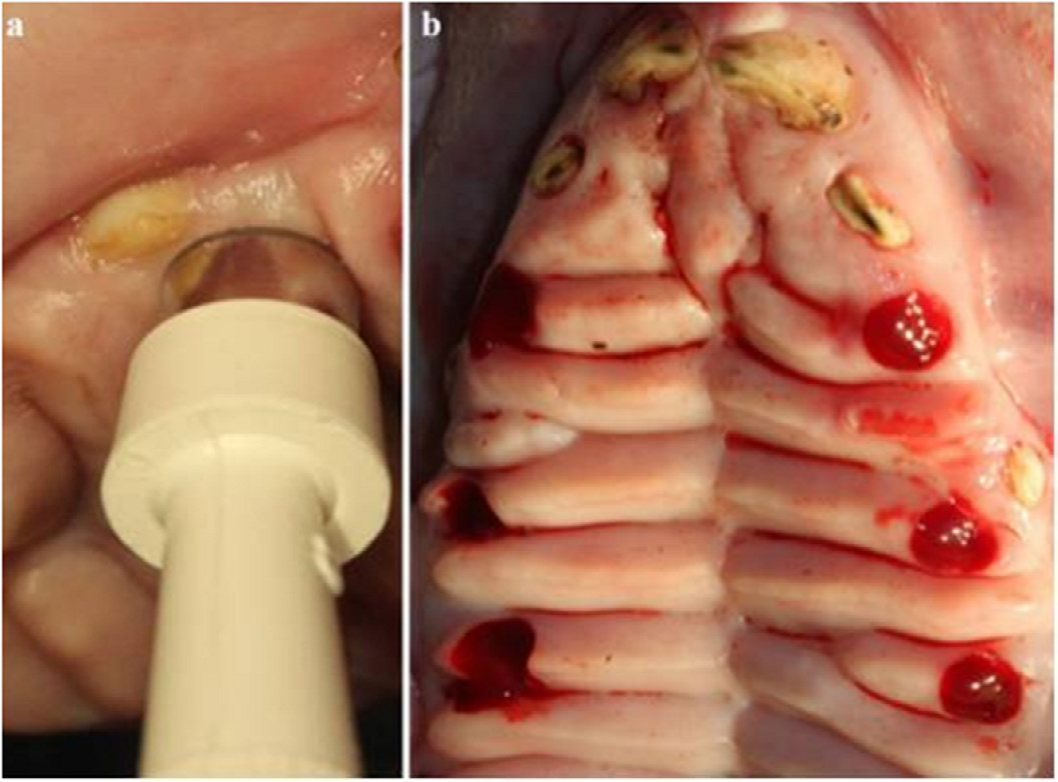
Creation of wounds on the palatal region

Three secondary wounds with a diameter of 10 mm and a depth of 2.5 mm were created on both sides of the palatine for a total of six circular wounds *per* animal, using the sterile punch biopsy knife according to the markings ( Figure 1 ).

### Topical ozone gas application

As ozone generator, an Ozonytron device (Ozonytron^®^, MIO International, GmbH, Germany) was used for the application of topical ozonetherapy. This generator has a glass probe; inside the probe, which is formed by a double glass camera, is a noble gases mixture that conducts and emits electromagnetic energy. When the tip of the probe comes in contact with the body, it emits energy around the treated area and splits environmental diatomic oxygen into singular atomic oxygen and ozone.

Three of the six wounds created on each pig were selected randomly and topical ozonetherapy was applied onto them through the ozone generator for ten days straight (test group). 60 μg/μl for 120-s ozone gas plasma (gingival healing stimulator mode of applicator) was applied, similar to the study of Akdeniz, et al. ^11^ (2018). No ozonetherapy was applied on to the other three wounds (control group).

**Figure 2:**
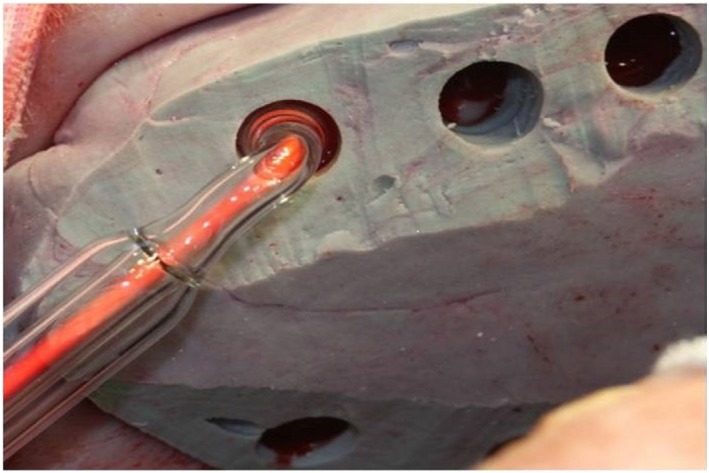
Application of ozone gas to test wounds

During the ozone application, the glass probe (10 mm in diameter) of the ozone generator was inserted through the perforations on the impression and the ozone was applied only to the selected zone. Enough space was created between perforation on the impression and probe for air circulation ( Figure 2 ).

The animals were given free access to water and food ( *ad libitum* ) but they were fed with soft food.

### Wound biopsy

Samples from all wounds and their edges were obtained with a sterile punch from the first wounds on day 3, from the second wounds on day 7, and from the third ones on day 10. Samples were immediately placed into containers with formaldehyde for histopathological and immuno-histochemical evaluations.

No animals were sacrificed during any period of our research.

### Histological procedures

After paraffin wax-embedding, sections (5 mm) were prepared across the middle of each wound and were stained with hematoxylin eosin (H&E).

### Histochemical procedures

This method is used to detect collagen fibers in tissues. Deparaffinized samples were placed again in Bouin's solution for 1 hour at 56°C. They were rinsed with running tap water for 5-10 minutes to remove the yellow color, stained in Weigert's iron hematoxylin working solution for 10 minutes, and rinsed in warm running tap water for 10 minutes. These rinsed samples were stained in a tricrom solution for 30 minutes, differentiated in a 5% glacial acetic acid solution for 2 minutes, washed in distilled water, and dehydrated and covered with xylene.

### Immuno-histochemical procedure

5 μm thick sections were assessed using immuno-histochemical staining to detect VEGF and TGF-β. Slides were deparaffinized and rehydrated respectively, using serial xylene baths, then through a graded series of ethanols. Sections were heated in a microwave oven with 0.01 M citrate buffer for 10 minutes to unmask the antigens. Endogenous peroxidase was blocked by a 10-minute treatment with 3% hydrogen peroxide at room temperature. The slides were washed with 0.1 M phosphate-buffered saline (PBS) for 5 minutes at pH 7.4 and then incubated with the primary antibodies overnight (TGF-β, Lot# K0910 mouse monoclonal IgG_1_ Santa Cruz Biotechnology Texas, USA and VEGF, lot# C0613 mouse monoclonal IgG_28_, Santa Cruz Biotechnology, Texas, USA) at room temperature. After washing with PBS (pH 7.4) for 10-15 minutes, the slides were incubated at room temperature for 10 minutes with an enhancer reagent (Biotinylated Goat Anti-Polyvalent, cat#TP-015-BN, Labvison Co, California, USA). After washing with PBS, slides were treated with polymer-HRP (HK 519-50K, BioGenex, Fremont, CA) for 30 minutes. Slides were washed with PBS and incubated with 3-amino-9-ethylcarbasole (AEC) (DAKO, Glostrup, Denmark) to obtain red staining of the immunoreactions. Finally, the slides were counterstained with Mayer's hematoxylin and cover slipped, to obtain positive control for TGF-β and VEGF sections of human upper stomach and liver respectively.

### Histological evaluation

Histological evaluation was done on samples stained with H&E and TRC under X20, X40 and X100 magnification. Inflammatory exudates, connective tissue and inflammatory cell density were evaluated in the wound area. Inflammatory cell density was scored according to the scoring system previously used by Ryu, et al. ^12^ (2012). The cell density was scored as follows: 0, no inflammation; 1, mild inflammation; 2, moderate inflammation; 3, intense inflammation. In this scoring system, the inflammatory responses based on the degree of neutrophil, histiocyte and lymphocyte infiltration, ulceration, fibrosis and granulation tissue were evaluated and scored using a scale of 0-3.

The trichrome-stained samples were evaluated to determine the epithelial differences between the test and control groups. The total width of the wound area and the amount of new epithelial tissue that proliferated centrally from the wound edges were calculated by using a 100-unit ocular grid. The calculated values were compared with each other and scored according to the epithelial grading method previously used by Innes, et al. ^13^ (2001). They were scored as follows: 0, no re-epithelialization; 1, less than 50% re-epithelialization; 2, more than 50% re-epithelialization; 3, completed re-epithelialization.

### Immuno-histochemical evaluation

The samples were evaluated by immuno-histochemical staining to detect VEGF and TGF-β under x200 magnification. Staining densities of the samples were scored as follows: 0, no staining; 1, mild staining; 2, moderate staining; 3, intense staining. The same scoring system was previously used by Ryu, et al. ^12^ (2012).

### Statistical evaluation

The data obtained in this study were analyzed using the Statistical Package for the Social Sciences (SPSS) 20 software.

Because the differences between the test and control groups were not included in the normal distribution, the Mann Whitney U test was used.

The Wilcoxon Sign Test was used for normal, nonscattering variables for testing the differences between TGF-β and VEGF values on the third, seventh, and tenth days in the test and control groups. The Spearman Correlation Analysis was used to investigate the relations among inflammation, TGF-β and VEGF values on days 3, 7 and 10 in the test and control groups.

Differences were considered significant as p<0.05.

## Results

### Histological results

**Figure 3:**
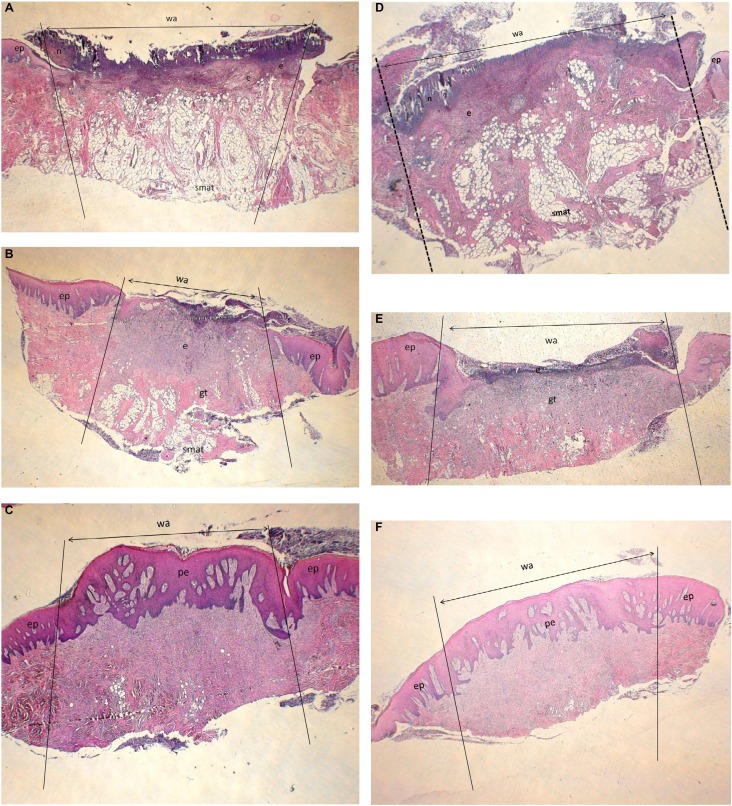
Histological results; Day 3 (A, D), Day 7 (B, E), Day 10 (C, F); test (A, B, C) ve Control (D, E, F) group. Appearance of wound area; (HE; x20) (wa: wound area; e: exudate; n: neutrophil; ep: epithelium; gt: granulation tissue; smat: submucosal adipose tissue; pe: proliferated epithelium)

In the test and control groups, it was observed that the surface epithelium and the lamina propria were completely separated from the wound area on day 3. The wound area had reached the submucosal adipose tissue ( Figure 3 A-F).

The wound area was filled with extensive fibrin exudates and neutrophilic infiltration. It was noted that the connective tissue adjacent to the wound base was rich in neutrophils. A mixed type of inflammation accompanied by plasma cells and lymphocytes was evident.

In the test and control groups, it was observed that the wound surface was covered with exudates on day 7. The wound area was filled with fibro vascular granulation tissue, and the inflammation had declined, compared to day 3.

Granulation tissue filling the wound area had consisted of capillary proliferation, inflammatory cells containing lymphocytes, histiocytes and polymorphonuclear leukocyte. No significant collagen production was observed in the wound area in the test or control groups, and surface epithelium had proliferated from the wound edges.

Exudate had disappeared from the wound surface, and the wound area was covered with surface epithelium in the test and control groups on day 10.

It was noted that there was an increase in the amount of collagen fibers in the exudates, unlike the 7^th^ day, and the lymphocytic infiltration had decreased when compared with the same day.

The inflammation values of days 3 and 7 were significantly higher than the values of day 10 in both groups (p<0.05).

**Figure 4:**
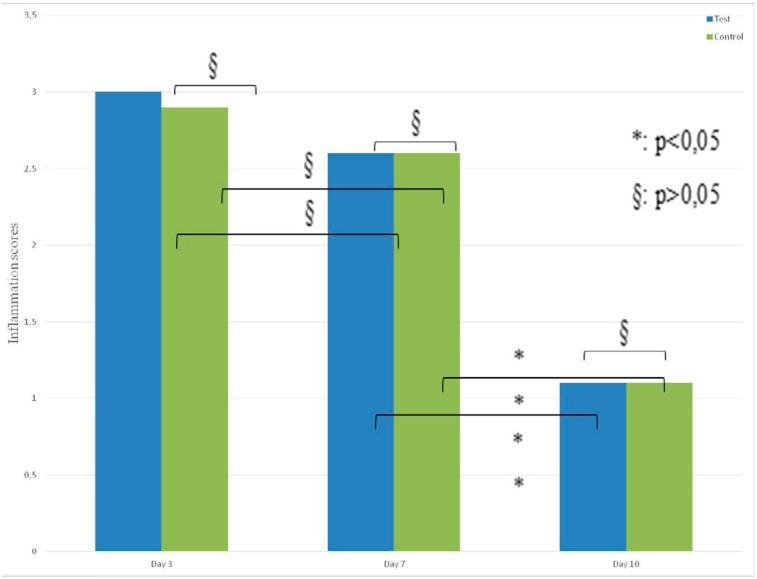
Inflammation scores of test and control groups on days 3, 7, 10 (p>0.05)

No statistically significant difference was observed when the inflammation scores of the test and control groups were compared with the scores recorded on days 3, 7, and 10 (p>0.05) ( Figure 4 ).

Epithelialization scores of samples taken on day 3 and 10 were the same for each day. For this reason, the scores of the samples from these days were not evaluated statistically. When the epithelialization scores of day 7 samples of the test and control groups were compared, no statistically significant difference was found (p<0.05).

### Immuno-histochemical results

#### VEGF

A positive VEGF was observed in the endothelial veins of the wound base of both groups on day 3.

VEGF staining was observed in the granulation tissue that was filling the wound area on day 7, but no staining was detected in the neutrophils around the exudates.

**Figure 5:**
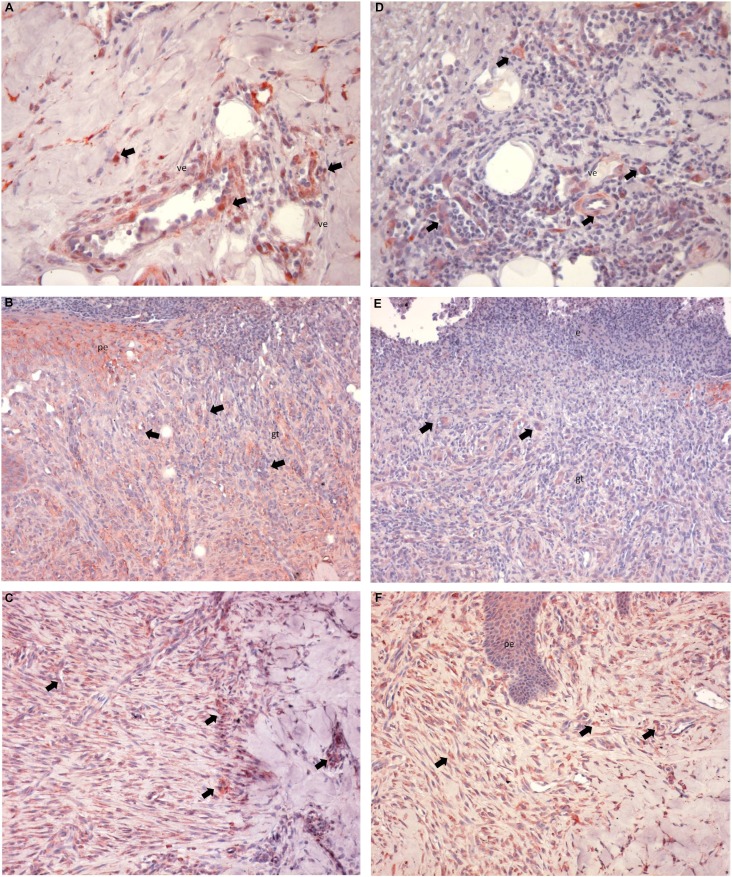
VEGF; Day 3 (A, D), Day 7 (B, E), Day 10 (C, F) and control (C, D) test (A, B, C) and Control (D, E, F) group, VEGF expression. (AEC; A, C, D, F: x200; B, E: x100 (ve: vascular endothelium; gt: granulation tissue; pe: proliferated epithelium; arrows: VEGF positive staining)

VEGF-positive staining was observed in the endothelial cells, fibroblasts, and in a small number of inflammatory cells in the wound area on day 10, as was observed on day 7 ( Figure 5 A-F).

When the changes in VEGF-staining intensity of the control group were evaluated on different days, there was no statistically significant difference between VEGF values on days 3 and 7 (p>0.05). The values taken on day 10 were significantly higher than the values taken on the other days (p<0.05).

When the changes in the VEGF-staining intensity of the test group were evaluated on different days, there was no statistically significant difference between VEGF values taken on days 3, 7, and 10 (p>0.05).

**Figure 6:**
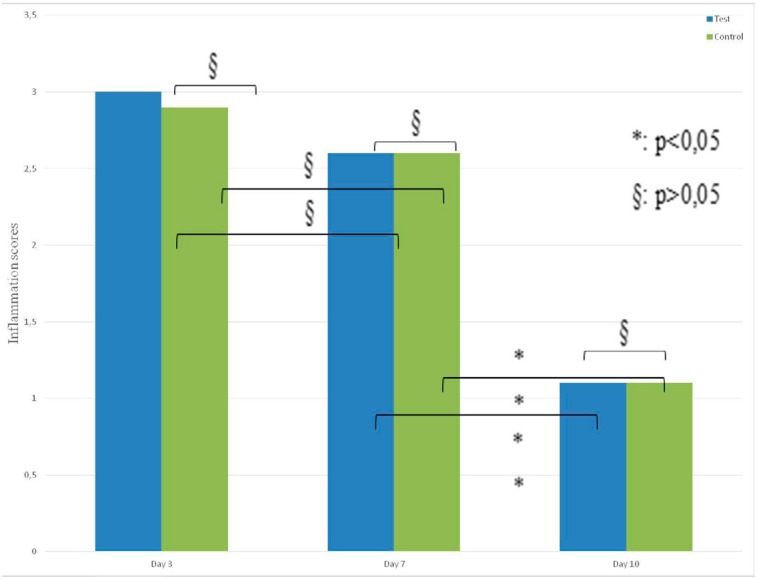
VEGF staining intensity between the test and control groups

When VEGF staining intensity of the test and control groups was compared with days 3, 7, and 10, the ones from the samples of the 3^rd^ and 7^th^ day in the test group were significantly higher than the control samples (p<0.05). No statistically significant differences were found between the test and control groups for VEGF values on day 10 (p>0.05) ( Figure 6 ).

#### TGF-β

On day 3, TGF-β staining was observed in both groups in the wound area, which was filled with exudates. Positive TGF-β was not detected in the vascular structures surrounding the submucosal connective tissue, or in the surrounding inflammatory cells.

**Figure 7:**
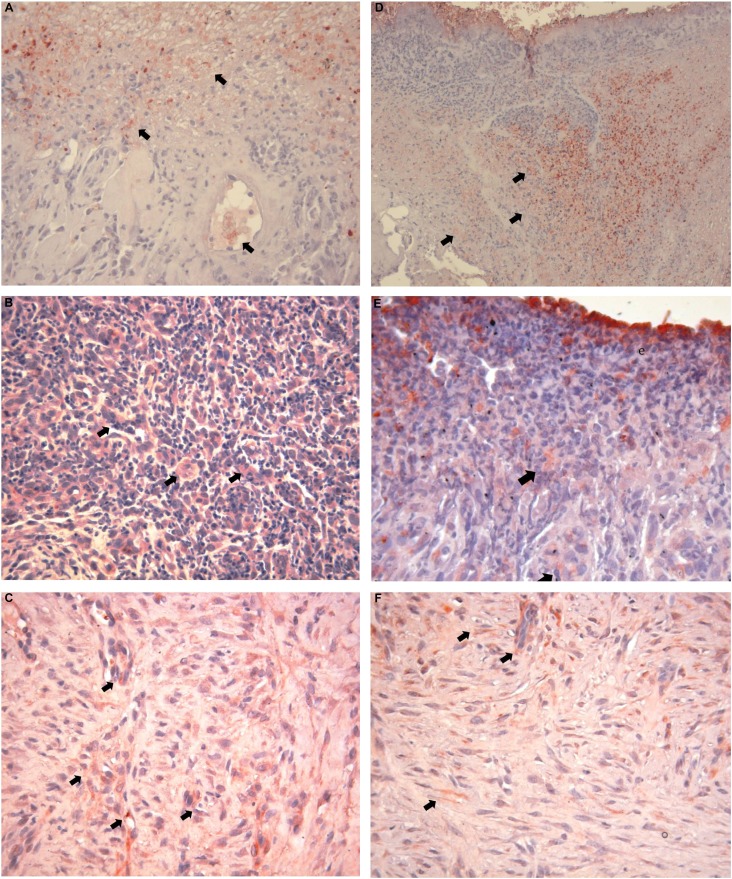
TGF-β; Day 3 (A,D), Day 7 (B, E), Day 10 (C, F) and control (C,D) test (A, B, C) and Control (D, E, F) group, TGF-β expression. (AEC; A, C, D, F: x200; B, E: x100) (e: exudate; arrows: TGF-β positive staining)

TGF-β staining was observed in the endothelial and inflammatory cells at the base of the exudates on days 7 and 10 ( Figure 7 A-F).

When the changes in TGF-β staining intensity of the control group taken on different days were evaluated, there was no statistically significant difference between TGF-β values taken on days 3, and 7 (p>0.05). The values taken on these days were significantly higher than the ones taken on day 10 (p <0.05).

When TGF-β staining intensity changes noted on different days within the test group were evaluated, there was no statistically significant difference between TGF-β values at days 3 and 7 (p>0.05). Values taken on day 3 were significantly higher than values taken on day 10 (p<0.05). There was no statistically significant difference between TGF-β values of days 7 and 10 (p>0.05).

**Figure 8:**
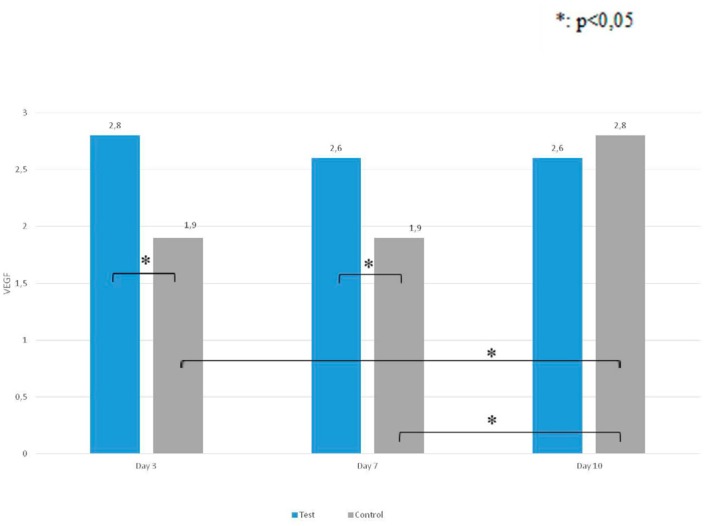
TGF-β staining intensity of the test and control groups

No statistically significant differences were observed (p>0.05) when TGF-β staining intensity within the test and control groups were compared on days 3, 7, and 10. Although TGF-β staining intensity was higher in the test group than in the control group on days 3, 7, and 10, this was not statistically significant ( Figure 8 ).

## Discussion

Because of its antimicrobial properties and positive effects on cutaneous injuries, one of the chemotherapeutic agents thought to be effective on surgical wounds in the oral cavity is ozone. However, there were no histological or immuno-histochemical studies evaluating the topical effect of ozonetherapy on wound healing around the gingival region. Accordingly, our study evaluated the healing effect of ozonetherapy produced by an ozone generator when applied to a created wound in the palatinal region.

The study was carried out using pigs because they have anatomical and physiological skin characteristics that are similar to humans ^9^ ^,^ ^14^ .

Prestudy power analysis determined that 7 animals should be randomized in order to attain an 80% statistical power, and we used 8 adult, healthy pigs in this experiment.

Days 3, 7 and 10 of the study were determined as examination period, according to a previous study by Kim, et al. ^9^ (2009).

It was decided that the control and experimental groups should consist of the same animals, and the samples taken on different days should be obtained from the same subject, in order to eliminate individual differences.

To our knowledge, there is no study evaluating the effect of ozonetherapy on wound healing using an ozone generator. The concentration of ozone gas plasma application was determined according to a study by Akdeniz, et al. ^11^ (2018).

Multiple endogenous growth factors, such as VEGF, TGF-β, FGF (Fibroblast Growth Factor) and PDGF (Platelet Derived Growth Factor), play an important role in early wound healing ^9^ . It may be important for healing to stimulate the expressions of endogenous growth factors at the local wound site ^15^ . Ozone increases endogenous growth factors, and it was thought that these could be used to evaluate the efficiency of ozone on early wound healing ^9^ ^,^ ^15^ . Because of these reasons, this study examines VEGF and TGF-β.

Yildirim, et al. ^16^ (2014) have evaluated the effects of ozone (ozone/oxygen mixture at a density of 0.7 g/kg) (OZONOSAN Photonic 1014, Hans GmbH Nordring & Iffezheim, Germany) and hyperbaric oxygen therapy (HBO) on an experimentally produced, secondary cutaneous wound healing process, and on inflammatory markers (tissue lipid peroxidation and inducible nitric oxide synthesis enzymes) in rats ^16^ . Rats were divided into three groups. The first group was treated with ozone, the second group with HBO, and the third group served as control. It was determined that all inflammatory markers decreased on days 1, 3, and 7 within the test and control groups. A significant decrease in inflammation was detected in both the Ozone and HBO groups, compared with the control group on days 3 and 7 (p<0.05). Similar to these results, inflammation scores in our study for both the test and control groups decreased on each of the three days. However, in our study, no statistically significant difference was found between test and control groups for inflammation scores on days 3, 7 and 10 (p>0.05).

Zhang, et al. ^15^ (2014) evaluated the efficacy of noninvasive oxygen-ozone therapy (52 μg/ml.) (Huma-zon Promedic, Germany) on secondary wound healing in 50 patients with type 2 diabetes and diabetic foot ulcers by using an immuno-histochemical analysis of VEGF and TGF-β in two groups: oxygen-ozone therapy (test) and no treatment (control). As a result of the study, it was seen that the VEGF and TGF-β values increased in all groups on days 7 and 11. Similarly, in our study, VEGF values of day 10 were significantly higher than the values of day 3 and 7 (p<0.05), however, there was no statistically significant difference between VEGF values on days 3 and 7 (p>0.05) in the control group. In the test group, when the changes of the VEGF staining intensity of the different days were evaluated, it was seen that VEGF values increased within days, but the increase was not statistically significant (p>0.05). No statistically significant difference was shown between TGF-β levels on days 3 and 7 (p>0.05), and the 3^rd^ and 7^th^ day values were significantly higher than the ones from the 10^th^ day (p<0.05) in the control group. In the test group, the 3^rd^ day values were significantly higher than the 10^th^ day values (p<0.05). There was no statistically significant difference between TGF-β levels on days 7 and 10 (p> 0.05). In the study by Zhang, et al. ^15^ (2014), VEGF levels on days 7 and 11 and TGF-β expression on day 11 were statistically higher in the ozone group than in the control group (P<0.001).

The study by Kim, et al. ^9^ (2009) was undertaken to evaluate the therapeutic effects of topical ozonated olive oil on acute cutaneous wound healing in a guinea pig mode. After creating full-thickness skin wounds on the backs of guinea pigs by using a 6 mm punch biopsy, the wound healing effect of the topically applied ozonated olive oil (ozone group) was examined, as compared to the pure olive oil (oil group) and no treatment (control group). The ozone group of guinea pigs had a significantly smaller wound size and residual wound area than the oil group, on days 5 (P<0.05) and 7 (P<0.01 and P<0.05) after wound surgery, respectively. Immuno-histochemical staining demonstrated the upregulation of PDGF, TGF-β and VEGF expressions in the ozone group on day 7, as compared with the oil group, but no statistical information was given.

In our study, when VEGF staining intensities of the test and control groups were compared on days 3, 7 and 10, the VEGF staining densities of the test group samples of the 3^rd^ and 7^th^ day were significantly higher than the control ones (p<0.05). There was no statistically significant difference between VEGF values of the test and control groups on day 10 (p>0.05). When the TGF-β staining intensity of the test and control groups were compared on days 3, 7 and 10, it was observed that the staining intensity of the test group was higher, but this difference was not statistically significant (p>0.05).

## Conclusion

Our study is the first research in literature that histologically and immuno-histochemically examines the efficacy of ozone gas on the secondary healing of wounds created in oral mucosal tissues. Following the results of our study, it can be speculated that topical ozonetherapy may have positive effect on the cascade of early tissue healing in the secondary palatal wounds by increasing the VEGF expression. However, further and longer histological and immuno-histochemical studies are needed to clarify the additional effects of topical ozonetherapy with different application methods on soft tissue healing.
